# Joint Source-Relay Optimization for MIMO Full-Duplex Bidirectional Wireless Sensor Networks with SWIPT

**DOI:** 10.3390/s19081827

**Published:** 2019-04-17

**Authors:** Dan Liu, Zhigang Wen, Xiaoqing Liu, Shan Li, Junwei Zou

**Affiliations:** Beijing Key Laboratory of Work Safety Intelligent Monitoring, School of Electronic Engineering, Beijing University of Posts and Telecommunications, Beijing 100876, China; dandanmessage@bupt.edu.cn (D.L.); xq0723@bupt.edu.cn (X.L.); lish01@ehualu.com (S.L.); buptzjw@bupt.edu.cn (J.Z.)

**Keywords:** beamforming, bidirectional wireless sensor network (BWSN), full duplex (FD), multiple-input multiple-output (MIMO), simultaneous wireless information and power transfer (SWIPT)

## Abstract

The simultaneous wireless information and power transfer (SWIPT) technique has been considered as a promising approach to prolong the lifetime of energy-constraint wireless sensor networks (WSNs). In this paper, a multiple-input multiple-output (MIMO) full-duplex (FD) bidirectional wireless sensor network (BWSN) with SWIPT is investigated. Based on minimum total mean-square-error (total-MSE) criterion, a joint optimization problem for source and relay beamforming and source receiving subject to transmitting power and harvesting energy constraints is established. Since this problem is non-convex, an iterative algorithm based on feasible point pursuit-successive convex approximation (FPP-SCA) is derived to obtain a local optimum. Moreover, considering the scenarios in which source and relay nodes equipped with the same and different numbers of antennas, a low-complexity diagonalizing design-based scheme is employed to simplify each non-convex subproblem into convex problems and to reduce the computational complexity. Numerical results of the total-MSE and bit error rate (BER) are implemented to demonstrate the performance of the two different schemes.

## 1. Introduction

Wireless sensor networks (WSNs) have attracted a significant amount of attention from researchers and have been widely employed in vast and varied areas, e.g., object tracking, habitat monitoring, military systems, and industrial areas [1,2,3]. However, in WSNs, the relay or sensor nodes are typically powered by batteries with finite capacities [4], which are difficult or impossible to replace or recharge in most cases. Thus, the energy supplies will limit the lifetime of WSNs. Saving on energy or prolonging the operation time of energy-constrained nodes has become an important research issue in WSNs. Traditionally, multi-input multi-output (MIMO) can provide an effective way for energy saving [5,6].

Recently, simultaneous wireless information and power transfer (SWIPT) is considered a promising energy-harvesting (EH) technique to solve the energy scarcity problem and to achieve perpetual communications in energy-constrained WSNs [7,8,9], which is extensively applied in the area. To date, two receiver architectures proposed in Reference [10], namely time switching (TS) and power splitting (PS), have been widely used for a colocated energy harvester and information decoder in SWIPT systems [11,12]. Compared with the TS structure periodically switching between the EH module and information decoding (ID) module, the PS design allows the receiver to complete EH and information processing in the same phase [13,14,15,16,17]. In Reference [14], the energy-efficient cooperative transmission problem for SWIPT and the power transfer in clustered WSNs was discussed, where the PS architecture was equipped with the receiver. In Reference [15], the joint transceiver design for full-duplex (FD) MIMO SWIPT systems with a PS mode was considered in order to minimize the mean square error (MSE). In Reference [16], the secrecy outage probability minimization problem for the decode-and-forward (DF) relay SWIPT systems with a PS scheme was analyzed. In Reference [17], the energy efficiency problem for SWIPT in a MIMO bidirectional amplify-and-forward (AF) relay network was formulated, where a receiver applied the PS scheme to harvest energy.

In the SWIPT context, conventionally, most networks are assumed to operate in the half-duplex (HD) communication mode [18,19,20,21,22]. Therein, in Reference [19], the performance of an HD bidirectional wireless sensor network (BWSN) with a TS EH strategy was analyzed. In Reference [20], a joint resource optimization scheme for the DF relay SWIPT cognitive sensor networks was proposed. In Reference [21], the authors investigated the joint source and the relay beamforming design in HD sensor networks with SWIPT. The joint source and relay precoding design for the HD bidirectional relay network (BRN) using a PS scheme was proposed in Reference [22]. However, in HD networks, communication nodes can either transmit or receive on a single frequency but not simultaneously [23]. Due to this characteristic, half of the spectrum resources are theoretically wasted. Recent advances suggest that the FD mode enables the concurrent transmission and reception of user signals over the same frequency band for which it can provide nearly double the improvement in spectral efficiency than HD [24]. Therefore, much interest has been turned to incorporating the networks into the FD [25,26,27,28,29,30,31,32]. Thereinto, in Reference [25], an FD MIMO one-way relay network (OWRN) aided by SWIPT was considered to solve the source and relay beamforming optimization problem using minimum mean-square-error (MSE) criterion. In Reference [26], a joint source and relay beamforming optimization for the FD one-way wireless sensor network (OWSN) with SWIPT using MSE minimization criterion was considered. In Reference [27], the transmission rate maximization problem for an FD OWRN powered by a wireless energy transfer was discussed. In Reference [28], the sum rate maximization problem for the AF FD relay-assisted MIMO one-way system was investigated, and with the consideration of self-interference aware FD relaying, an alternating optimization (AO) method was devised. In Reference [29], the authors designed the source and relay precoders for a MIMO FD OWRN with SWIPT-enabled destination to optimize the end-to-end performance in residual loop-interference environments. In Reference [30], the hardware impairments of the FD AF OWRN was considered and an optimization problem was established to maximize the signal to a distortion-plus-noise ratio under relay and source transmit power constraints.

Nonetheless, motivated by the benefit of reducing the waste of extra-channel resources and achieving a higher spectral efficiency than the one-way communication [33,34,35], the bidirectional communication has attracted considerable interest, and much more researches have tended to adopt bidirectional communication in the FD. In the literature [36], the joint optimization of transmit and receive beamforming for relays to maximize the achievable sum-rate in the FD BRN system with a PS scheme was considered. However, to the best of our knowledge, a joint source-relay design based on a total-MSE minimization in MIMO FD BWSN with SWIPT has not yet been studied.

In this paper, a MIMO FD BWSN with PS is presented. With the consideration of processing self-interference, different from References [15,27,28,29,30], we choose to use the one presented in Reference [37]. The merit of the proposed network lies in the considerably high spectral efficiency, providing a cost-effective and perpetual power supply for WSNs and an uninterrupted transmission of information. The contributions are summarized as follows. First, for the sensor system model, contrary to Reference [36], the two source nodes are also equipped with multiple transmitter-receiver antennas for signal transmission and reception, and the multiple data streams transmitting scenarios are considered. Second, a joint optimization problem for source and relay beamforming and source receiving based on the total-MSE minimization is formulated. Third, to cope with the primal nonconvex problem, a feasible point pursuit-successive convex approximation (FPP-SCA)-based iterative algorithm is exploited. Finally, to reduce the computational complexity, a low-complexity diagonalizing method-based algorithm is introduced to simplify each non-convex subproblem into convex problems directly. In terms of the existing approach [25], the generalized singular value decomposition (GSVD) is discussed, and the scenarios in which source and relay nodes equipped with the same and different number of antennas are both discussed. The numerical results show a good performance and validate our analysis.

The remainder of the paper is organized as follows. Section 2 proposes the system model, including the sensor nodes deployment and optimization model. Section 3 focuses on the scheme design. The numerical results are presented and discussed in Section 4. Finally, the conclusions are presented in Section 5.

***Notation:*** Throughout this paper, scalar variables are expressed by lowercase italic letters, vectors are represented by boldface lowercase letters, and matrices are denoted by boldface uppercase letters. $C^{M\times N}$ denotes an M×N matrix with complex entries. Tr(·), ${\left(\cdot \right)}^{T}$, ${\left(\cdot \right)}^{H}$, ${\left(\cdot \right)}^{-1}$, ${\left(\cdot \right)}^{*}$, and $∥\cdot ∥$ are the trace, transpose operation, conjugate transpose operation, inverse operation, conjugate transpose operation, and the bound norm of a vector. $\sum_{i=1}^{M}\left(\cdot \right)$ stands for the sum from 1 to *M*. $∼CN\left(x,\sigma^{2}\right)$ represents a complex Gaussian distributed variable with a mean *x* and covariance σ. vec(·) and mat(·) are the matrix vectorization operator and the corresponding inverse operation, respectively. E[·] signifies the expectation of the random variables in the bracket.

## 2. System Model

This paper aims to jointly design the transmitters of the source and relay and the receivers of the source in the FD BWSN with SWIPT. We adopt a three-node sensor system consisting of two sources and a relay and assume that the sources are equipped with the PS receiver and that the relay applies an AF scheme. Without a loss of generality, we suppose that the energy conversion efficiency at the PS receiver is 100 percent and that the PS ratio is fixed.

**Figure 1:**
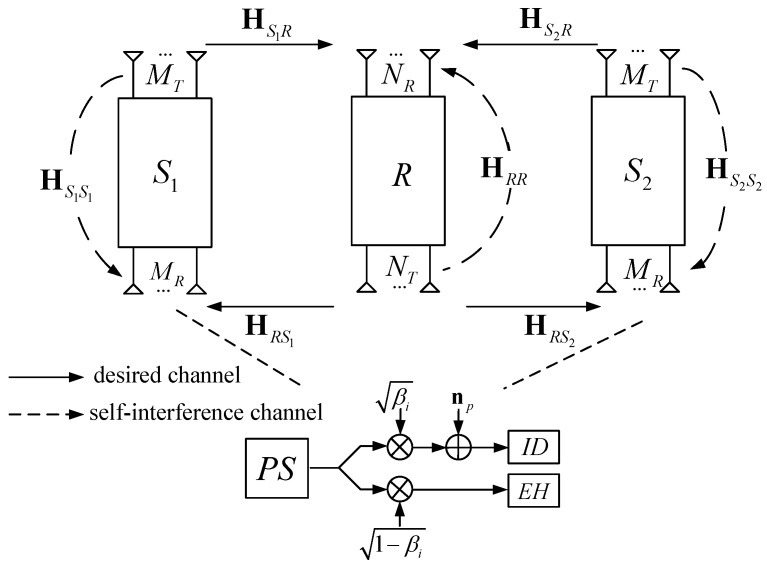
The system model of the multi-input multi-output (MIMO) full-duplex (FD) bidirectional wireless sensor network (BWSN) with energy harvesting (EH).

The considered three-node MIMO BWSN with SWIPT consists of two sources: $S_{1}$ and $S_{2}$ both equipped with $M_{T}>1$ transmit antennas and $M_{R}>1$ receive antennas. $S_{1}$ and $S_{2}$ decode the information, harvest the energy by PS, and exchange information with the help of the single AF relay node *R* with $N_{T}>1$ transmit antennas and $N_{R}>1$ receive antennas, as shown in Figure 1. All nodes are assumed to operate in FD mode, which means they transmit and receive data at the same time and frequency.

Let $H_{S_{i}R}\in C^{N_{R}\times M_{T}}$ and $H_{RS_{i}}\in C^{M_{R}\times N_{T}}$ denote channel matrices from $S_{i}$’s transmit antennas to *R*’s receive antennas and that from *R*’s transmit antennas to $S_{i}$’s receive antennas, respectively. We assume that all channels are statistically independent, reciprocal in the incoming and outgoing directions, and slowly time-varying quasi-static flat Rayleigh fading. Moreover, the self-interference channels at the corresponding nodes are represented as $H_{S_{1}S_{1}}\in C^{M_{R}\times M_{T}}$, $H_{S_{2}S_{2}}\in C^{M_{R}\times M_{T}}$, and $H_{RR}\in C^{N_{R}\times N_{T}}$.

Meanwhile, in our system, the two source nodes $S_{1}$ and $S_{2}$ are set far apart so that the direct link between them is assumed to be ignored. Moreover, we suppose that the perfect channel state information (CSI) is available at each node [38,39,40] and that the transmit power of the two sources are equal.

For a further analysis, the node deployment and optimization models are presented as follows.

### 2.1. Node Deployment

At time instant *n*(n>1), $M_{T}$ data streams $s_{i}\left[n\right]\in C^{M_{T}\times 1}$ with a normalized power are transmitted through the beamformer $F_{i}\in C^{M_{T}\times M_{T}}$$\left(i\in \left\{1,2\right\}\right)$ from $S_{i}$ simultaneously and the relay *R* forwards its received signal $y_{R}\left[n\right]\in C^{N_{R}\times 1}$ after multiplying it by a beamforming matrix $F_{r}\in C^{N_{T}\times N_{R}}$. In practice, a τ(τ⩾1)-symbol processing delay is unavoidable at *R* when it processes the received signals.

Accordingly, in time slot *n*, the received signal $y_{R}\left[n\right]$ at *R* can be expressed as

(1) $$y_{R}\left[n\right]=\sum_{i=1}^{2}H_{S_{i}R}F_{i}s_{i}\left[n\right]+H_{RR}x_{R}\left[n\right]+n_{r}\left[n\right],$$

where $n_{r}\left[n\right]∼CN\left(0,\sigma_{r}^{2}I_{N_{r}}\right)$ represents the additive white Gaussian noise (AWGN) at *R*.

Assuming that τ is kept small and that the SI can be cancelled perfectly or almost perfectly with the knowledge of the signal transmitted by the relay itself [37], the transmitted signal $x_{R}\left[n\right]\in C^{N_{T}\times 1}$ at *R* can be written as

(2) $$x_{R}\left[n\right]=F_{r}\left(y_{R}\left[n-\tau \right]-H_{RR}x_{R}\left[n-\tau \right]\right).$$

Substituting Equation (1) into Equation (2), the overall relay output can be given by

(3) $$x_{R}\left[n\right]=F_{r}\left(\sum_{i=1}^{2}H_{S_{i}R}F_{i}s_{i}\left[n-\tau \right]+n_{r}\left[n-\tau \right]\right).$$

The signal received by $S_{i}$, $i\in \left\{1,2\right\}$ can be written as

(4) $$\begin{array}{cc} {\hat{y}}_{S_{i}}\left[n\right]= & H_{RS_{i}}\left[F_{r}\left(H_{S_{\bar{i}}R}F_{\bar{i}}\left[n-\tau \right]s_{\bar{i}}\left[n-\tau \right]+H_{S_{i}R}F_{i}s_{i}\left[n-\tau \right]\right)\right]+H_{S_{i}S_{i}}x_{S_{i}}\left[n\right]+{\hat{n}}_{i}\left[n\right], \end{array}$$

where, the same as below, $\bar{i}=2$ if i=1 and vice versa. ${\hat{n}}_{i}\left[n\right]$ is the equivalent noise vector representing ${\hat{n}}_{i}\left[n\right]=H_{RS_{i}}F_{r}n_{r}\left[n-\tau \right]+n_{i}\left[n\right]$, where $n_{i}\left[n\right]$ denotes an AWGN at source nodes $S_{i}$ with $n_{i}\left[n\right]∼CN\left(0,\sigma_{S_{i}}^{2}I_{M_{R}}\right)$.

For simplicity, we assume that the full channel state information (CSI) is known and that both $S_{1}$ and $S_{2}$ know their own transmitted signals; thus, the SI at $S_{i}$ herein can be cancelled. After subtracting the back-propagated self-interference term $H_{RS_{i}}F_{r}H_{S_{i}R}F_{i}s_{i}\left[n-\tau \right]$ from Equation (4), the received signal at $S_{i}$ becomes

(5) $${\hat{y}}_{S_{i}}\left[n\right]=H_{RS_{i}}F_{r}\left(H_{S_{\bar{i}}R}F_{\bar{i}}s_{\bar{i}}\left[n-\tau \right]+n_{r}\left[n-\tau \right]\right)+n_{i}\left[n\right].$$

To implement SWIPT, a portion $\beta_{i}\in \left(0,1\right)$ of the signal power is applied to Equation (5), which splits ${\hat{y}}_{S_{i}}\left[n\right]$ into two parts, $\beta_{i}$ portion for ID and the remaining $\left(1-\beta_{i}\right)$ portion for EH. Then, the signals for ID at each source node can be represented as

(6) $$y_{S_{i}}\left[n\right]=\sqrt{\beta_{i}}H_{RS_{i}}F_{r}H_{S_{\bar{i}}R}F_{\bar{i}}s_{\bar{i}}\left[n-\tau \right]+n_{i}^{{}^{\prime }}\left[n,\tau \right],$$

where $n_{i}^{{}^{\prime }}\left[n,\tau \right]=\sqrt{\beta_{i}}\left(H_{RS_{i}}F_{r}n_{r}\left[n-\tau \right]+n_{i}\left[n\right]\right)+n_{p}\left[n\right]$ denotes the equivalent noise vector and $n_{p}\left[n\right]∼CN\left(0,\sigma_{p}^{2}I_{M_{R}}\right)$ is the AWGN caused by the power splitter.

At the EH side, we have

(7) $$\zeta_{i}\left(1-\beta_{i}\right)Tr\left[H_{RS_{i}}F_{r}RF_{r}^{H}H_{RS_{i}}^{H}+\sigma_{S_{i}}^{2}I_{M_{R}}\right]⩾{\bar{e}}_{i},$$

where $\zeta_{i}\in \left(0,1\right]$ is the energy conversion efficiency at the energy harvester. It is assumed that for $\zeta_{i}=1$, here, ${\bar{e}}_{i}$ represents the minimum power that should be harvested at $S_{i}$ and $R=\sum_{i=1}^{2}H_{S_{i}R}F_{i}F_{i}^{H}H_{S_{i}R}^{H}+\sigma_{r}^{2}I_{N_{R}}$.

Moreover, $F_{i}$ and $F_{r}$ should satisfy the transmitting power constraints, that is,

(8) $$Tr\left(F_{i}F_{i}^{H}\right)⩽p_{i},\;\;Tr\left(F_{r}RF_{r}^{H}\right)⩽p_{r},\;$$

where $p_{i}$ and $p_{r}$ are the maximum transmit power supplied by $S_{i}$ and *R*, respectively.

Since channels in our system are memoryless, we can define that $y_{S_{i}}≜y_{S_{i}}\left[n\right]$, $n_{i}^{{}^{\prime }}≜n_{i}^{{}^{\prime }}\left[n,\tau \right]$, $s_{\bar{i}}≜s_{\bar{i}}\left[n-\tau \right]$, and $n_{r}≜n_{r}\left[n-\tau \right]$, and we assume that $\sigma_{p}^{2}\ll \sigma_{S_{i}}^{2}$. Thus, (6) can be reformulated as

(9) $$y_{S_{i}}=\sqrt{\beta_{i}}\left(H_{RS_{i}}F_{r}H_{S_{\bar{i}}R}F_{\bar{i}}s_{\bar{i}}+H_{RS_{i}}F_{r}n_{r}+n_{i}\right).$$

### 2.2. Optimization Model

Considering the harvested energy and transmit power constraints, i.e., Equations (7) and (8), the optimization model to minimize the total-MSE of the whole system and to find the optimal source and relay beamformer and the source receiver is described in this section. The objective function and the problem are separately discussed below.

Using Equation (9), the MSE of $S_{i}$ can be given by

(10) $$\begin{array}{cc} J_{i}= & E\left[{∥W_{i}y_{S_{i}}-s_{\bar{i}}\;∥}_{2}^{2}\right]\\ = & Tr\{W_{i}J_{i_{B}}J_{i_{B}}^{H}W_{i}^{H}+\beta_{i}\sigma_{S_{i}}^{2}W_{i}W_{i}^{H}\\ & -2Re\left(W_{i}J_{i_{B}}\right)+\sigma_{r}^{2}W_{i}J_{i_{A}}J_{i_{A}}^{H}W_{i}^{H}+I_{M_{R}}\}, \end{array}$$

where $W_{i}\in C^{M_{R}\times M_{R}}$ is the linear receiver at $S_{i}$, $E[s_{i}s_{\bar{i}}^{H}]=0$, $E\left[s_{i}s_{i}^{H}\right]=1$, $J_{i_{A}}=\sqrt{\beta_{i}}H_{RS_{i}}F_{r}$, $J_{{\bar{i}}_{C}}=H_{S_{\bar{i}}R}F_{\bar{i}}$, and $J_{i_{B}}=J_{i_{A}}J_{{\bar{i}}_{C}}$.

Given the MSE of $S_{i}$, fixing $\beta_{i}$, a joint source and relay beamforming and source-receiving optimization problem based on the total-MSE with transmit power constraints and an energy-harvesting constraint can be formulated as

(11a) $$\min_{F_{r},W_{i},F_{i}}\;\;\;J_{1}+J_{2}\;\;\;\;\;\;\;\;$$

(11b) $$s.t.\;\;\;\;\;Tr\left(F_{i}F_{i}^{H}\right)⩽p_{i}\;\;\;\;\;\;$$

(11c) $$\;Tr\left(F_{r}RF_{r}^{H}\right)⩽p_{r}\;\;\;\;$$

(11d) $$\left(1-\beta_{i}\right)Tr\left[H_{RS_{i}}F_{r}RF_{r}^{H}H_{RS_{i}}^{H}+\sigma_{S_{i}}^{2}I_{M_{R}}\right]⩾{\bar{e}}_{i}.$$

## 3. Scheme Design

Considering the problem Equation (11) is non-convex and multivariate, the iterative algorithms based on FPP-SCA and a low-complexity diagonalizing are employed in this section.

### 3.1. Iterative Algorithm Based on FPP-SCA

Since the problem in Equation (11) is non-convex and basically intractable, in this section, an iterative algorithm based on FPP-SCA [41] is proposed to decouple the primal problem into four subproblems corresponding to four variables: $W_{i}$, $F_{r}$, $F_{1}$, and $F_{2}$, and to solve them alternately. At each iteration, one variable is optimized while keeping the other fixed. Starting from Equation (12), the $W_{i}$ is optimized, and then, the $F_{r}$ is optimized by using Equation (14), following this, Equation (16) (actually two subproblems) is formulated to optimize $F_{1}$ and $F_{2}$ separately. Finally, the four subproblems are solved, and the four variables are optimized. Details are given below.

First, with $F_{i}$ and $F_{r}$ fixed, the receiver $W_{i}$ is first optimized. As $W_{i}$ is only involved in $J_{i}$, the optimal $W_{i}^{opt}$ can be derived using $\partial J_{i}/\partial W_{i}^{*}=0$, which yields

(12) $$W_{i}^{opt}=J_{i_{B}}^{H}R_{wi}^{-1},$$

where $R_{wi}=\left(J_{i_{B}}J_{i_{B}}^{H}+\sigma_{r}^{2}J_{i_{A}}J_{i_{A}}^{H}+\beta_{i}\sigma_{S_{i}}^{2}I_{M_{R}}\right)$.

#### 3.1.1. Optimization of Relay Beamformer $F_{r}$

Then, the optimization of $F_{r}$ with a fixed $F_{i}$ and $W_{i}$ is discussed. According to [42] (p. 77),

(13) $$Tr\left(\mathrm{ABCD}\right)={\left(vec(D^{T})\right)}^{T}\left(C^{T}⊗A\right)vec\left(B\right),$$

where A, B, C, and D are arbitrary matrices with compatible dimensions, ⊗ is the Kronecker product, and vec(·) represents the matrix vectorization operator.

To guarantee the feasibility of Equation (11), the feasible region is relaxed and approximated by adding slacks $s\in R^{2}$ to the non-convex constraint of Equation (11d), and the positive slack variables and slack penalty are used in Equation (11a). Then, the original problem can be recast as

(14a) $$\begin{array}{cc} \min_{f_{r}}\; & f_{r}^{H}Z_{r}f_{r}-Q_{r}^{H}f_{r}-f_{r}^{H}Q_{r}+C_{r}+\lambda ∥s∥\;\; \end{array}$$

(14b) $$\begin{array}{cc} s.t. & \;\;\;\;f_{r}^{H}Q_{ir}f_{r}⩽-\xi_{i}+s_{m}\;\; \end{array}$$

(14c) $$\begin{array}{c} \;\;\;\;f_{r}^{H}Qf_{r}⩽p_{r}\;\; \end{array}$$

(14d) $$\begin{array}{c} \;\;\;\;s_{m}⩾0,\;\;m=1,2,\;\; \end{array}$$

where $∥\cdot ∥$ can be any vector norm, $∥s∥$ denotes the slack penalty term, and λ⩾1 is the trade-off between the original objective function and $∥s∥$. Besides, $f_{r}=vec\left(F_{r}\right)$, $Z_{r}=Z_{r1}+Z_{r2}$, $Q_{r}=Q_{r1}+Q_{r2}$, $C_{r}=C_{r1}+C_{r2}$, $Z_{ri}=\beta_{i}{\left(J_{{\bar{i}}_{C}}J_{{\bar{i}}_{C}}^{H}+\sigma_{r}^{2}I_{N_{R}}\right)}^{T}⊗\left(H_{RS_{i}}^{H}W_{i}^{H}W_{i}H_{RS_{i}}\right)$, $Q_{ri}=\sqrt{\beta_{i}}vec\left(J_{{\bar{i}}_{C}}^{H}H_{RS_{i}}^{H}W_{i}^{H}\right)$, $C_{ri}=Tr\left(\beta_{i}\sigma_{S_{i}}^{2}W_{i}W_{i}^{H}+I_{M_{R}}\right)$, $Q=I_{N_{R}}⊗R^{T}$, $Q_{ir}=-H_{RS_{i}}^{H}H_{RS_{i}}⊗R^{T}$, and $\xi_{i}=\frac{{\bar{e}}_{i}}{\left(1-\beta_{i}\right)}-Tr\left(\sigma_{S_{i}}^{2}I_{M_{R}}\right)$.

Since $Q_{ir}$ is negative, Equation (14) is non-convex. To tackle this subproblem, we define $g\left(f_{r}\right)=f_{r}^{H}Q_{ir}f_{r}$ and assume that a center point $z_{r}\in C^{N\times 1}$, $N=N_{T}\times N_{R}$ is given. Introducing $\tilde{g}\left(f_{r}\right)≜2Re\left(z_{r}^{H}Q_{ir}f_{r}\right)-z_{r}^{H}Q_{ir}z_{r}$, Theorem 1 can be established and proved.

**Theorem** **1.** *$\tilde{g}$ satisfies the following properties: (i) $\tilde{g}\left(z_{r}\right)=g\left(z_{r}\right)$; (ii) $\tilde{g}\left(f_{r}\right)\geq g\left(f_{r}\right)$; and (iii) $\partial \tilde{g}\left(f_{r}\right)/\partial f_{r}{{|}_{f_{r}=z_{r}}=\partial g\left(f_{r}\right)/\partial f_{r}|}_{f_{r}=z_{r}}$.*

**Proof.**  Substituting $z_{r}$ into $\tilde{g}\left(f_{r}\right)$ and $g(f_{r})$, (i) can be easily certified. For (ii), $Q_{ir}⪯0$, ${\left(f_{r}-z_{r}\right)}^{H}Q_{ir}\left(f_{r}-z_{r}\right)⩽0$ always holds, which shows $g\left(f_{r}\right)=f_{r}^{H}Q_{ir}f_{r}⩽f_{r}^{H}Q_{ir}z_{r}+z_{r}^{H}Q_{ir}f_{r}-z_{r}^{H}Q_{ir}z_{r}=2Re\left(z_{r}^{H}Q_{ir}f_{r}\right)-z_{r}^{H}Q_{ir}z_{r}=\tilde{g}\left(f_{r}\right)$, property (ii) is proved. For (iii), the derivatives can be computed as $\partial \tilde{g}\left(f_{r}\right)/\partial f_{r}{|}_{f_{r}=z_{r}}={\left(z_{r}^{H}Q_{ir}\right)}^{T}$ and $\partial g\left(f_{r}\right)/\partial f_{r}{|}_{f_{r}=z_{r}}={\left(z_{r}^{H}Q_{ir}\right)}^{T}$; therefore, property (iii) is proved. □

Replacing $g(f_{r})$ with $\tilde{g}(f_{r})$, Equation (14) can be rewritten as

(15a) $$\begin{array}{cc} \min_{f_{r}}\; & f_{r}^{H}Z_{r}f_{r}-Q_{r}^{H}f_{r}-f_{r}^{H}Q_{r}+C_{r}+\lambda ∥s∥\;\; \end{array}$$

(15b) $$\begin{array}{cc} s.t.\; & 2Re\left(z_{r}^{H}Q_{ir}f_{r}\right)⩽z_{r}^{H}Q_{ir}z_{r}-\xi_{i}+s_{m}\;\; \end{array}$$

(15c) $$\begin{array}{c} \;\;\;\;f_{r}^{H}Qf_{r}⩽p_{r}\;\; \end{array}$$

(15d) $$\begin{array}{c} \;\;s_{m}⩾0,\;\;m=1,2.\;\; \end{array}$$

Equation (15) can be efficiently solved using the modeling language YALMIP [43] and the generic conic programming solver SeDuMi [44]. A new approximated problem can be built and solved when the optimal solution of Equation (15) becomes the new center point, that is, $z_{r}=f_{r}^{*}$. Based on Theorem 1, Equation (14) can be solved.

#### 3.1.2. Optimization of Source Beamformer $F_{i}$

Similarly, $F_{i}$ can be optimized given $F_{r}$ and $W_{i}$. According to Equation (13), the original problem in Equation (11) can be transformed into Equation (16)

(16a) $$\begin{array}{cc} \min_{f_{i}}\; & f_{i}^{H}Q_{i}f_{i}-q_{i}^{H}f_{i}-f_{i}^{H}q_{i}+C_{i}+\lambda ∥s∥\;\;\;\;\;\; \end{array}$$

(16b) $$\begin{array}{cc} s.t.\; & \;\;\;\;\;\;\;f_{i}f_{i}^{H}⩽p_{i}\;\;\;\;\;\; \end{array}$$

(16c) $$\begin{array}{c} \;\;\;\;f_{i}^{H}Q_{1i}f_{i}⩽p_{r}-Tr\left(Q_{f_{i}}\right)\;\;\;\;\;\; \end{array}$$

(16d) $$\begin{array}{c} \;\;\;\;\;f_{i}^{H}Q_{{2i}_{D}}^{{}^{\prime }}f_{i}⩽\Delta_{i_{D}}\;\;\;\;\;\; \end{array}$$

(16e) $$\begin{array}{c} \;\;\;\;\;f_{i}^{H}Q_{{2i}_{\bar{D}}}^{{}^{\prime }}f_{i}⩽\Delta_{i_{\bar{D}}}\;\;\;\;\;\; \end{array}$$

(16f) $$\begin{array}{c} \;\;\;\;s_{m}⩾0,\;\;m=1,2,\;\;\;\;\; \end{array}$$

where $f_{i}=vec\left(F_{i}\right)$, $Q_{i}=\beta_{\bar{i}}\left(I_{M_{T}}⊗A_{i}A_{i}^{H}\right)$, $q_{i}=\sqrt{\beta_{\bar{i}}}vec{\left(A_{i}\right)}^{H}$, $A_{i}=W_{\bar{i}}H_{RS_{\bar{i}}}F_{r}H_{S_{i}R}$, D=1, $\bar{D}=2$, $Q_{{2i}_{D}}^{{}^{\prime }}=-I_{M_{T}}⊗Q_{iD}^{H}Q_{iD}$, $Q_{iD}=H_{RS_{D}}F_{r}H_{S_{i}R}$, $\Delta_{i_{D}}=Tr\left(Q_{{Si}_{D}}+\sigma_{S_{D}}^{2}I_{M_{R}}\right)-\frac{{\bar{e}}_{D}}{\left(1-\beta_{D}\right)}+s_{D}$, $Q_{{Si}_{D}}=H_{RS_{1}}F_{r}Q_{Ci}F_{r}^{H}H_{RS_{1}}^{H}$, $Q_{Ci}=J_{{\bar{i}}_{C}}J_{{\bar{i}}_{C}}^{H}+\sigma_{r}^{2}I_{N_{R}}$, $Q_{1i}=I_{M_{T}}⊗H_{S_{i}R}^{H}F_{r}^{H}F_{r}H_{S_{i}R}$, $Q_{f_{i}}=F_{r}J_{{\bar{i}}_{C}}J_{{\bar{i}}_{C}}^{H}F_{r}^{H}+\sigma_{r}^{2}F_{r}F_{r}^{H}$,    $C_{i}=Tr\left(C_{i_{D}}+C_{i_{\bar{D}}}+\sigma_{S_{i}}^{2}W_{i}W_{i}^{H}+\sigma_{S_{\bar{i}}}^{2}W_{\bar{i}}W_{\bar{i}}^{H}+2I_{M_{T}}\right)-\sqrt{\beta_{i}}Tr\left(R_{W}\right)-\sqrt{\beta_{i}}\left(R_{W}^{H}\right)+Tr\left(R_{W}R_{W}^{H}\right)$, $R_{W}=W_{i}J_{i_{B}}$, and $C_{i_{D}}=\sigma_{r}^{2}\beta_{D}W_{D}H_{RS_{D}}F_{r}F_{r}^{H}H_{RS_{D}}^{H}W_{D}^{H}I_{M_{R}}$.

The optimized $f_{1}$ and $f_{2}$ can be separately obtained from Equation (16) using a similar FPP-SCA method foresaid.

#### 3.1.3. Summarization of the Proposed Algorithm

Based on the FPP-SCA algorithm presented above, the iterative algorithm is summarized as Algorithm 1.

**Algorithm 1** An alternating optimization algorithm based on a feasible point pursuit-successive convex approximation (FPP-SCA)
**1. Initialize**
Define $\beta_{i}=0.5$, $F_{r}=\sqrt{\frac{p_{r}}{Tr(R)}}I_{N_{R}}$, and $F_{i}=\sqrt{\frac{p_{i}}{M_{T}}}I_{M_{T}}$.
**2. Iterative updating**
(1) Update $W_{i}$ using Equation (12) with a fixed $F_{i}$ and $F_{r}$.(2) Update $F_{r}$ by solving Equation (14) with a fixed $F_{i}$ and $W_{i}$.a. Set k=0 and $vec(F_{r})$ as the initial point $z_{r}^{0}$. b. Solve Equation (15) at the *k*th iteration for k⩾0 to yield the optimal solution $f_{r}^{k}$. c. Let $z_{r}^{k+1}=f_{r}^{k}$ and k=k+1. d. Until convergence, let $F_{r}=mat\left(z_{r}^{k+1}\right)$.(3) Update $F_{i}$ by solving Equation (16) with a fixed $F_{r}$ and $W_{i}$, following similar steps in (2).
**3. Until convergence**


The Algorithm 1 is convergent based on the following Property 1.

**Property** **1.** *The iterative algorithm based on FPP-SCA is convergent.*

**Proof.**  In the *k*th iteration of the proposed algorithm, we first compute $F_{r}^{\left[k\right]}$ with the given $F_{1}^{\left[k-1\right]}$, $F_{2}^{\left[k-1\right]}$, and $W_{i}^{\left[k-1\right]}$. Since the optimal solution $F_{r}^{\left[k\right]}$ can be achievable with CVX, where CVX is a Matlab-based available convex programming toolbox [45], we can discover that the objective value corresponding to $F_{r}^{\left[k\right]}$, $F_{1}^{\left[k-1\right]}$, $F_{2}^{\left[k-1\right]}$, and $W_{i}^{\left[k-1\right]}$ is no greater than that to $F_{r}^{\left[k-1\right]}$, $F_{1}^{\left[k-1\right]}$, $F_{2}^{\left[k-1\right]}$, and $W_{i}^{\left[k-1\right]}$. Similarly, $F_{1}^{\left[k\right]}$ is no larger than that to $F_{1}^{\left[k-1\right]}$, $F_{2}^{\left[k\right]}$ is no larger than that to $F_{2}^{\left[k-1\right]}$, and $W_{i}^{\left[k\right]}$ is optimally solved and the objective value is descendent. Consequently, the objective value of the original problem monotonically decreases and is lower-bounded by zero, which verifies the convergence of Algorithm 1. □

### 3.2. Low-Complexity Diagonalizing Design

However, the main drawback of the proposed FPP-SCA algorithm is the high computational complexity. In order to overcome this shortcoming, a low-complexity algorithm using the channel parallelization (CP) technique [22], namely the generalized singular value decomposition (GSVD) and SVD, is applied.

In this section, we assume that $M_{T}=M_{R}=M$ and $N_{T}=N_{R}=N$ for simplicity and focus on the scenarios where N⩾M.

#### 3.2.1. Channel Parallelization

Substituting Equation (12) into Equation (10) and employing

(17) $$E^{-1}-{\left(F^{-1}E+I\right)}^{-1}E^{-1}={\left(E+F\right)}^{-1},$$

where E and F are arbitrary matrices and I is the identity matrix, the function $J_{i}$ can be simplified as

(18) $$J_{i}=Tr\left[{\left(I_{M_{T}}+J_{i_{B}}^{H}Z_{1i}^{-1}J_{i_{B}}\right)}^{-1}\right],$$

where $Z_{1i}=\sigma_{r}^{2}J_{i_{A}}J_{i_{A}}^{H}+\beta_{i}\sigma_{S_{i}}^{2}I_{M_{R}}$.

Applying GSVD on the uplink channel matrix pair $\left\{H_{S_{1}R}^{H},H_{S_{2}R}^{H}\right\}$ and SVD on the downlink channels $H_{dl}={\left[H_{RS_{1}}^{T},H_{RS_{2}}^{T}\right]}^{T}$, we can obtain

(19) $$H_{S_{1}R}=R_{h}\Sigma_{h_{1}}U_{h_{1}}^{H},\;\;H_{S_{2}R}=R_{h}\Sigma_{h_{2}}U_{h_{2}}^{H},\;$$

(20) $$H_{RS_{1}}=R_{dl1}\Sigma_{dl}U_{dl}^{H},\;\;H_{RS_{2}}=R_{dl2}\Sigma_{dl}U_{dl}^{H},$$

where $R_{h}\in C^{N\times N}$, $U_{h_{i}}\in C^{M\times M}$, $R_{dl}\in C^{2M\times 2M}$, and $U_{dl}\in C^{N\times N}$ are four unitary matrices; $\Sigma_{h_{1}}={\left(0_{(N-M)\times M}^{T},\Lambda_{h_{1}}^{T}\right)}^{T}$; $\Sigma_{h_{2}}={\left(\Lambda_{h_{2}}^{T},0_{(N-M)\times M}^{T}\right)}^{T}$; $\Sigma_{dl}={\left(\Lambda_{dl}^{T},0_{(2M-N)\times N}^{T}\right)}^{T}$; $\Lambda_{h_{i}}$ and $\Lambda_{dl}$ are M×M and N×N nonnegative diagonal matrices; $R_{dl1}=R_{dl}\left(1:M,1:N\right)$; $R_{dl2}=R_{dl}\left(M+1:N,1:N\right)$ for N>M; and $R_{dl1}={\tilde{R}}_{dl1}=R_{dl}\left(1:M,1:M\right)$, $R_{dl2}={\tilde{R}}_{dl2}=R_{dl}\left(M+1:2M,1:M\right)$ for N=M.

In order to parallelize the channels in Equations (19) and (20), the relay and source beamformers $F_{r}$ and $F_{i}$ can be proposed as

(21) $$F_{r}=U_{dl}\Lambda_{r}R_{h}^{-1},\;\;F_{i}=U_{h_{i}}\Lambda_{i}V_{F_{i}}^{H},$$

where $\Lambda_{r}$ and $\Lambda_{i}$ are N×N and M×M nonnegative diagonal matrices, respectively.

Substituting Equations (19)–(21) into Equation (18), the resultant objective function $J_{i}^{*}$ becomes

(22) $$\begin{array}{cc} J_{i}^{*}= & Tr\left[{\left(I_{M_{T}}+\beta_{i}\Lambda_{\bar{i}}^{H}\Sigma_{h_{\bar{i}}}^{H}\Lambda_{r}^{H}\Lambda_{dl}^{H}{\left(\sigma_{r}^{2}\beta_{i}\Lambda_{dl}\Lambda_{r}\Lambda_{B_{h}}\Lambda_{r}^{H}\Lambda_{dl}^{H}+\Lambda_{B_{i}}\beta_{i}\sigma_{S_{i}}^{2}I_{M_{R}}\right)}^{-1}\Lambda_{dl}\Lambda_{r}\Sigma_{h_{\bar{i}}}\Lambda_{\bar{i}}\right)}^{-1}\right], \end{array}$$

where $\Lambda_{B_{h}}$ and $\Lambda_{B_{i}}$ are two diagonal matrices containing the (k,k)th entries of $B_{h}$ and $B_{i}$, $B_{h}={\left(R_{h}R_{h}^{H}\right)}^{-1}$, and $B_{i}={\left(R_{dli}^{H}R_{dli}\right)}^{-1}$ for N>M, $B_{i}={\left({\tilde{R}}_{dli}^{H}{\tilde{R}}_{dli}\right)}^{-1}$ for N=M.

Substituting Equations (19)–(21) into each of the constraints in Equation (11), the original problem can be expressed as

(23a) $$\min_{\Lambda_{r},\Lambda_{i}}\;\;\;\;\;\;J_{1}^{*}+J_{2}^{*}\;\;\;\;\;\;\;\;\;$$

(23b) $$s.t.\;\;\;Tr\left(\Lambda_{i}\Lambda_{i}^{H}\right)⩽p_{i}\;\;\;\;\;\;\;$$

(23c) $$\;\;\;\;\;\;\left(1-\beta_{i}\right)Tr\left[\Gamma_{G}+\sigma_{S_{i}}^{2}I_{M_{R}}\right]⩾{\bar{e}}_{i},\;\;\;\;\;\;\forall i\;\;\;\;\;\;\;$$

(23d) $$\;\;\;\;\;\;Tr\left[\Lambda_{r}\left(\Gamma +B_{h}\sigma_{r}^{2}I_{N_{R}}\right)\Lambda_{r}^{H}\right]⩽p_{r},\;\;\;\;\;\;\;$$

where $\Gamma_{G}=B_{i}^{-1}\Lambda_{dl}\Lambda_{r}\left(\Gamma +B_{h}\sigma_{r}^{2}I_{N_{R}}\right)\Lambda_{r}^{H}\Lambda_{dl}^{H}$ and $\Gamma =\Sigma_{h_{1}}\Lambda_{1}\Lambda_{1}^{H}\Sigma_{h_{1}}^{H}+\Sigma_{h_{2}}\Lambda_{2}\Lambda_{2}^{H}\Sigma_{h_{2}}^{H}$. To solve the nonconvexity caused by Equation (23c), we propose Theorem 2 as follows.

**Theorem** **2.** *The left side of the energy-harvesting constraint in Equation* (23c) *can be replaced by its lower-bound $\left(1-\beta_{i}\right)Tr\left[\Lambda_{\Gamma_{G}}+\sigma_{S_{i}}^{2}I_{M_{R}}\right]$.*

**Proof.**  We take N>M as an example to illustrate the proof procedure and the optimization problem. Expanding the left side of Equation (23c), defining $A=R_{dli}\Lambda_{dl}\Lambda_{r}$, and ignoring the constant matrix $\sigma_{r}^{2}I_{N_{R}}$, the part $Tr\left[B_{i}^{-1}\Lambda_{dl}\Lambda_{r}B_{h}\Lambda_{r}^{H}\Lambda_{dl}^{H}\right]$ becomes

(24) $$Tr\left[AB_{h}A^{H}\right]=Tr\left[AA^{H}B_{h}\right]=Tr\left[CB_{h}\right].$$

Define the (i,j) entry of $CB_{h}$ as ${\left(CB_{h}\right)}_{ij}=\sum_{k=1}^{M}c_{ik}b_{h_{kj}}$; then, we have $Tr\left(CB_{h}\right)⩾Tr\left(C\Lambda_{B_{h}}\right)$ based on the relationship $\sum_{i=1}^{M}\sum_{k=1}^{M}c_{ik}b_{h_{ki}}⩾\sum_{i=1}^{M}c_{ii}b_{h_{ii}}$. Following a similar procedure, the lower-bound of $Tr\left[B_{i}^{-1}\Lambda_{dl}\Lambda_{r}\Gamma \Lambda_{r}^{H}\Lambda_{dl}^{H}\right]$ can be expressed as $Tr\left[\Lambda_{B_{i}}^{-1}\Lambda_{dl}\Lambda_{r}\Gamma \Lambda_{r}^{H}\Lambda_{dl}^{H}\right]$.

Then, Theorem 2 is proved. □

By using Theorem 2, Equation (23) can be reformulated as

(25a) $$\begin{array}{cc} \min_{\Lambda_{r},\Lambda_{i}}\; & \;\;\;\;\;J_{1}^{*}+J_{2}^{*}\;\;\; \end{array}$$

(25b) $$\begin{array}{cc} s.t.\; & \;\;\;Tr\left(\Lambda_{i}\Lambda_{i}^{H}\right)⩽p_{i}\;\;\; \end{array}$$

(25c) $$\begin{array}{c} \left(1-\beta_{i}\right)Tr\left[\Lambda_{\Gamma_{G}}+\sigma_{S_{i}}^{2}I_{M_{R}}\right]⩾{\bar{e}}_{i},\;\;\;\;\;\;\forall i\;\;\; \end{array}$$

(25d) $$\begin{array}{c} Tr\left[\Lambda_{r}\left(\Gamma +\Lambda_{B_{h}}\sigma_{r}^{2}I_{N_{R}}\right)\Lambda_{r}^{H}\right]⩽p_{r},\;\;\; \end{array}$$

where $\Lambda_{\Gamma_{G}}=\Lambda_{B_{i}}^{-1}\Lambda_{dl}\Lambda_{r}\left(\Gamma +\Lambda_{B_{h}}\sigma_{r}^{2}I_{N_{R}}\right)\Lambda_{r}^{H}\Lambda_{dl}^{H}$.

#### 3.2.2. Alternating Optimization of $F_{r}$ and $F_{i}$

In this section, an iterative approach is utilized to convert the multivariate non-convex problem in Equation (25) into three convex subproblems. We first study how to optimize $\Lambda_{r}$ with a fixed $\Lambda_{i}$, and then, the alternating optimization of $\Lambda_{1}$ and $\Lambda_{2}$ is performed with a given $\Lambda_{r}$.

1. Optimization of $\Lambda_{r}$

Using Equation (17), $J_{i}^{*}$ can be simplified and rewritten as

(26) $$J_{i}^{*}=M-Tr\left\{\left[\beta_{i}\Lambda_{\bar{i}}^{H}\Sigma_{h_{\bar{i}}}^{H}D_{i}^{-1}\Sigma_{h_{\bar{i}}}\Lambda_{\bar{i}}-{\mathrm{MSE}}_{i}^{*}\right]\right\},$$

where ${\mathrm{MSE}}_{i}^{*}=\beta_{i}\left(\Lambda_{\bar{i}}^{H}\Sigma_{h_{\bar{i}}}^{H}D_{i}^{-1}\Lambda_{\bar{i}}\Sigma_{h_{\bar{i}}}\right)\left[\frac{1}{\beta_{i}\sigma_{S_{i}}^{2}}\Lambda_{r}^{H}\Lambda_{dl}^{H}\Lambda_{dl}\right.$ ${\left.\Lambda_{r}\Lambda_{B_{i}}^{-1}D_{i}+I_{M}\right]}^{-1}$ and $D_{i}=\beta_{i}\Sigma_{h_{\bar{i}}}\Lambda_{\bar{i}}\Lambda_{\bar{i}}^{H}\Sigma_{h_{\bar{i}}}^{H}+\sigma_{r}^{2}\beta_{i}\Lambda_{B_{h}}$.

Since $\Lambda_{r}$ exists in ${\mathrm{MSE}}_{i}^{*}$ only, the problem of minimizing $J_{1}^{*}+J_{2}^{*}$ is equivalent to that of minimizing ${\mathrm{MSE}}_{1}^{*}+{\mathrm{MSE}}_{2}^{*}$. Defining $a_{in}$, $a_{h_{i}n}$, $a_{rn}$, $a_{dn}$, $\lambda_{B_{i}n}$, and $\lambda_{B_{h}n}$ as the *n*th diagonal element of $\Lambda_{i}$, $\Lambda_{h_{i}}$, $\Lambda_{r}$, $\Lambda_{dl}$, $\Lambda_{B_{i}}$, and $\Lambda_{B_{h}}$, respectively, ${\mathrm{MSE}}_{i}^{*}$ can be given by

(27) $${\mathrm{MSE}}_{1}^{*}=\sum_{n=1}^{M}\frac{\beta_{1}^{2}\sigma_{S_{1}}^{2}a_{2n}^{2}a_{h_{2}n}^{2}\lambda_{B_{1}n}}{\left(a_{rn}^{2}a_{dn}^{2}\lambda_{1n}+\lambda_{B_{1}n}\beta_{1}\sigma_{S_{1}}^{2}\right)\lambda_{1n}},$$

(28) $${\mathrm{MSE}}_{2}^{*}=\sum_{n=\varsigma }^{N}\frac{\beta_{2}^{2}\sigma_{S_{2}}^{2}a_{1n}^{2}a_{h_{1}n}^{2}\lambda_{B_{2}n}}{\left(a_{rn}^{2}a_{dn}^{2}\lambda_{2n}+\lambda_{B_{2}n}\beta_{2}\sigma_{S_{2}}^{2}\right)\lambda_{2n}},$$

where ς=N−M+1, $\lambda_{in}=\beta_{i}a_{h_{\bar{i}n}}^{2}a_{\bar{i}n}^{2}+\sigma_{r}^{2}\beta_{i}\lambda_{B_{h}n}$.

Moreover, we define $\phi_{in}=a_{rn}^{2}a_{h_{\bar{i}n}}^{2}a_{\bar{i}n}^{2}$, $\theta_{rn}=a_{rn}^{2}\lambda_{B_{h}n}$, $\Phi_{in}=\phi_{in}a_{dn}^{2}\lambda_{B_{i}n}^{-1}$, and $\Theta_{rn}=\theta_{rn}\lambda_{B_{i}n}^{-1}a_{dn}^{2}$. Accordingly, the problem related to $\Lambda_{r}$ can be described as

(29a) $$\begin{array}{cc} \min_{a_{rn}^{2}}\; & \;\;\;{\mathrm{MSE}}_{1}^{*}+{\mathrm{MSE}}_{2}^{*}\; \end{array}$$

(29b) $$\begin{array}{cc} s.t.\; & \;\;\;\left(1-\beta_{i}\right)\left[\Lambda_{r_{c}}\right]⩾{\bar{e}}_{i}\; \end{array}$$

(29c) $$\begin{array}{c} \sum_{n=1}^{M}\phi_{1n}+\sum_{n=\varsigma }^{N}\phi_{2n}+\sum_{n=1}^{N}\sigma_{r}^{2}\theta_{rn}⩽p_{r}\; \end{array}$$

(29d) $$\begin{array}{c} \;\;\;\;\;a_{rn}⩾0,\; \end{array}$$

where $\Lambda_{r_{c}}=\sum_{n=1}^{M}\Phi_{1n}+\sigma_{S_{i}}^{2}I_{M}+\sum_{n=\varsigma }^{N}\Phi_{2n}+\sum_{n=1}^{N}\sigma_{r}^{2}\Theta_{rn}$.

2. Optimization of $\Lambda_{i}$

Similarly, the solution for $a_{in}$ can be described in the following scalar form

(30a) $$\begin{array}{cc} \min_{a_{in}^{2}}\; & \;\;\;\;J_{1}^{□}+J_{2}^{□}\;\;\; \end{array}$$

(30b) $$\begin{array}{cc} s.t.\; & \;\;\sum_{n=1}^{M}a_{in}^{2}⩽p_{i},\;\;\;\;\;\;\;a_{in}⩾0\;\;\; \end{array}$$

(30c) $$\begin{array}{c} \;\;\;\left(1-\beta_{i}\right)\left[\Lambda_{r_{c}}\right]⩾{\bar{e}}_{i}\;\;\; \end{array}$$

(30d) $$\begin{array}{c} \sum_{n=1}^{M}\phi_{1n}+\sum_{n=\varsigma }^{N}\phi_{2n}+\sum_{n=1}^{N}\sigma_{r}^{2}\theta_{rn}⩽p_{r},\;\;\; \end{array}$$

where $J_{1}^{□}=\sum_{n=1}^{M}{\left(1+\frac{\beta_{1}a_{2n}^{2}a_{rn}^{2}a_{h_{2}n}^{2}a_{dn}^{2}}{\sigma_{r}^{2}\beta_{1}a_{dn}^{2}a_{rn}^{2}\lambda_{B_{h}n}+\beta_{1}\sigma_{S_{1}}^{2}\lambda_{B_{1}n}}\right)}^{-1}$ and $J_{2}^{□}=\sum_{n=\varsigma }^{N}\left(1+\right.$${\left.\frac{\beta_{2}a_{1n}^{2}a_{rn}^{2}a_{h_{1}n}^{2}a_{dn}^{2}}{\sigma_{r}^{2}\beta_{2}a_{dn}^{2}a_{rn}^{2}\lambda_{B_{h}n}+\beta_{2}\sigma_{S_{2}}^{2}\lambda_{B_{2}n}}\right)}^{-1}$.

By proving

(31) $$\frac{\partial^{2}{\mathrm{MSE}}_{i}^{*}}{\partial^{2}a_{rn}^{2}}=\frac{2{\beta_{i}}^{2}\sigma_{S_{i}}^{2}a_{\bar{i}n}^{2}a_{h_{\bar{i}n}}^{4}\lambda_{B_{i}n}a_{dn}^{2}\lambda_{a_{i}n}}{{\left(a_{rn}^{2}a_{dn}^{2}\lambda_{a_{i}n}+\lambda_{B_{i}n}\beta_{i}\sigma_{S_{i}}^{2}\right)}^{3}}⩾0,$$

and

(32) $$\frac{\partial^{2}J_{i}^{□}}{\partial^{2}a_{\bar{i}n}^{2}}=\frac{2b_{in}{\left(\beta_{i}a_{rn}^{2}a_{h_{\bar{i}n}}^{2}a_{dn}^{2}\right)}^{2}}{{\left(b_{in}+\beta_{i}a_{\bar{i}n}^{2}a_{rn}^{2}a_{h_{\bar{i}n}}^{2}a_{dn}^{2}\right)}^{3}}⩾0,$$

where for i=1, 1⩽n⩽M and for i=2, N−M+1⩽n⩽N and $b_{in}=\sigma_{r}^{2}\beta_{i}a_{dn}^{2}a_{rn}^{2}\lambda_{B_{h}n}+\beta_{i}\sigma_{S_{i}}^{2}\lambda_{B_{i}n}$, we can indicate that Equations (29) and (30) are convex for $a_{rn}^{2}$ and $a_{in}^{2}$. Then, the optimal solution can be obtained by CVX directly.

#### 3.2.3. Summarization of the Proposed Algorithm

The low-complexity algorithm based on CP method depicted above is summarized as Algorithm 2.

**Algorithm 2** The low-complexity algorithm based on the channel parallelization (CP) method
**1. Channel decomposition**
Decompose the channel pairs $\left\{H_{S_{1}R}^{H},H_{S_{2}R}^{H}\right\}$ and $\left\{H_{RS_{1}},H_{RS_{2}}\right\}$ using Equations (19) and (20).
**2. Initialization**
Define $\beta_{i}=0.5$, $F_{r}=\sqrt{\frac{p_{r}}{\mathrm{Tr}(\Psi_{R})}}I_{N}$, and $F_{i}=\sqrt{\frac{p_{i}}{M_{T}}}I_{M_{T}}$, where $\Psi_{R}=R_{h}\Sigma_{h_{1}}\Lambda_{1}\Lambda_{1}^{H}\Sigma_{h_{1}}^{H}R_{h}^{H}+R_{h}\Sigma_{h_{2}}\Lambda_{2}\Lambda_{2}^{H}\Sigma_{h_{1}}^{H}R_{h}^{H}+\sigma_{r}^{2}I_{N_{R}}$.
**3. Iterative updating**
(1) Update $W_{i}$ using Equation (12) with a fixed $F_{i}$ and $F_{r}$.(2) Update $F_{r}$ with a fixed $F_{i}$ and $W_{i}$.a. Update $\Lambda_{r}$ using $a_{rn}$ by solving Equation (29). b. Substitute $\Lambda_{r}$ into $F_{r}=U_{dl}\Lambda_{r}R_{h}^{-1}$.(3) Update $F_{i}$ with a fixed $F_{r}$ and $W_{i}$.a. Update $\Lambda_{1}$ and $\Lambda_{2}$ using $a_{1n}$ and $a_{2n}$ by solving Equation (30) separately. b. Substitute $\Lambda_{1}$ and $\Lambda_{2}$ into $F_{i}=U_{h_{i}}\Lambda_{i}V_{F_{i}}^{H}$.
**4. Until convergence**


Algorithm 2 is convergent based on the following Property 2.

**Property** **2.** *The low-complexity algorithm based on the CP method is convergent.*

**Proof.**  In the *k*th iteration of the proposed algorithm, we first compute $F_{r}^{\left[k\right]}$ with the given $F_{1}^{\left[k-1\right]}$, $F_{2}^{\left[k-1\right]}$, and $W_{i}^{\left[k-1\right]}$. Since the optimal solution $F_{r}^{\left[k\right]}$ can be achievable with CVX, we discover that the objective value corresponding to $F_{r}^{\left[k\right]}$, $F_{1}^{\left[k-1\right]}$, $F_{2}^{\left[k-1\right]}$, and $W_{i}^{\left[k-1\right]}$ is no greater than that to $F_{r}^{\left[k-1\right]}$, $F_{1}^{\left[k-1\right]}$, $F_{2}^{\left[k-1\right]}$, and $W_{i}^{\left[k-1\right]}$, which means the objective value is descendent. Consequently, the objective value of the original problem monotonically decreases and is lower-bounded by zero, which verifies the convergence of Algorithm 2. □

## 4. Numerical Results and Discussion

In order to analyze the performance of the proposed algorithms, the following simulations are conducted. Fifty random Rayleigh fading channels are generated, and the pathloss exponent is set to 2. The variances of noises are assumed as $\sigma_{r}^{2}=\sigma_{S_{i}}^{2}=\sigma^{2}$, the transmit powers are set as $p_{i}=18E_{s}$ and $p_{r}=12E_{s}$, and the signal noise ratio (SNR) is calculated from $SNR=10log_{10}\left(E_{s}/\sigma^{2}\right)$, where $E_{s}$ is the power of signal. Meanwhile, the energy-harvesting requirement ${\bar{e}}_{i}=0.1$. N=4, M=2 and N=2, M=2 are both considered, and the data stream S=2. Moreover, the carrier frequency of the system is given by $f_{c}=5$ GHz. Four schemes are simulated: 1. The unaided scheme, which means that the beamformers are set as initial matrices; 2. the proposed FPP-SCA scheme; 3. the proposed low-complexity scheme; and 4. the semidefinite relaxation (SDR) scheme [46] used in the previous literature. In order to show the impact of noise, the impact of different values of β, and the number of antennas, we do the corresponding simulations.

**Figure 2:**
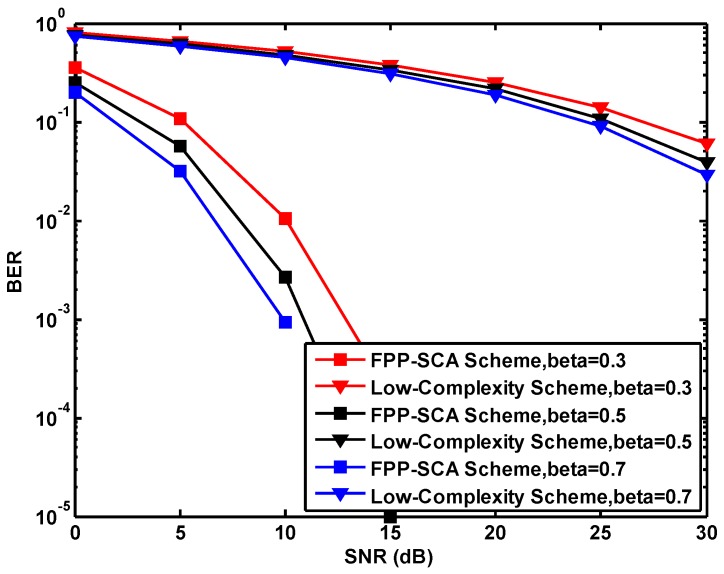
The bit error rate (BER) versus signal noise ratio (SNR) for the proposed schemes under different β.

Figure 2 and Table 1 show the performance under different β for the proposed FPP-SCA scheme and the Low-Complexity scheme. From the simulation results, obviously, a larger β leads to a higher system performance for both schemes, since more signals can be used for decoding the information in the receiver shown in Equation (6). In order to make the comparision with the existing works, we choose to use β=0.5.

**Figure 3:**
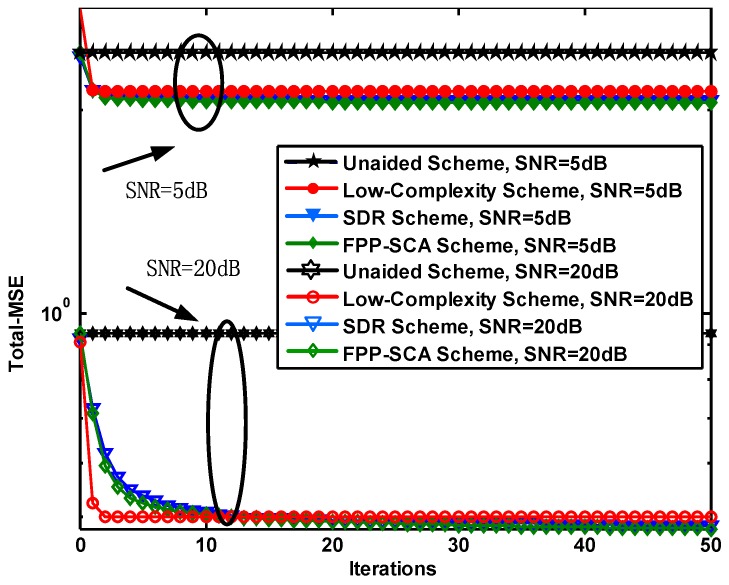
The total mean square error (total-MSE) versus the iterations for N=M=2.

The convergence property of different schemes is evaluated in Figure 3, where the total-MSE is plotted versus the iterations ranging from 0 to 50 in SNR=5 dB and SNR=20 dB when N=M=2. From Figure 3, as the increment in the number of iterations, the FPP-SCA scheme always converges slower and requires more iterations for a convergence as SNR increases than the proposed low-complexity one. Furthermore, the FPP-SCA scheme exhibits a better performance than the low-complexity one for different SNRs when the curve converges. Meanwhile, comparing the FPP-SCA and conventional SDR scheme, we can find that the SDR scheme always converges slower than the FPP-SCA one and that the MSE of it is always higher than that of the FPP-SCA one (e.g., 2.07 vs. 2.04 for SNR=5 dB and 0.50 vs. 0.48 for SNR=20 dB) under 50 iterations, which implies an advantage of the proposed FPP-SCA scheme.

It can be claimed that the proposed FPP-SCA scheme has a lower level of total-MSE while it has a higher iteration complexity than the low-complexity counterpart.

**Figure 4:**
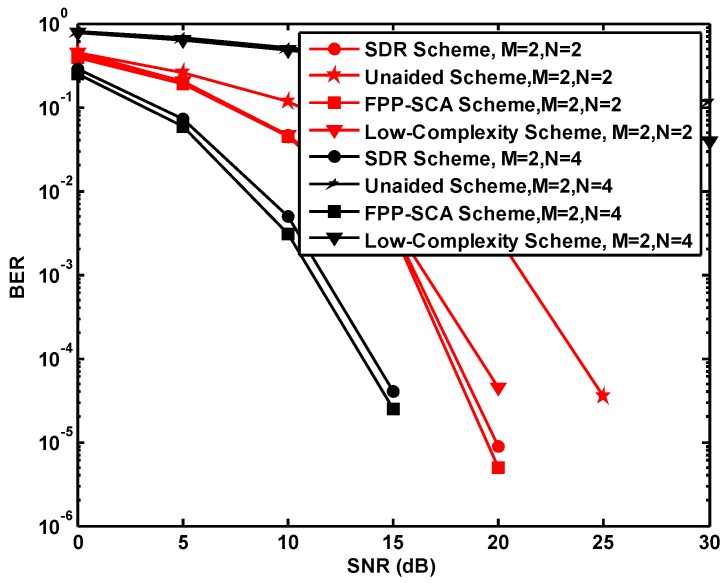
BER versus SNR for 50 iterations.

The performance comparison of different schemes is indicated in Figure 4, Table 2 and Table 3, where in Figure 4, the bit error rate (BER) is plotted against SNR ranging from 0 dB and 30 dB under conditions N=M=2 and N=4,M=2 with respect to 50 iterations. From the results illustrated in Figure 4, obviously as the SNR increases, the BER decreases for all schemes. Meanwhile, in both conditions of antenna, the FPP-SCA one is the best in terms of the performance of all schemes, which increases the performance 0.003 compared with the Unaided Scheme, $0.4\times 10^{-4}$ compared with the Low-Complexity Scheme, and $4.0\times 10^{-6}$ compared with the SDR Scheme for N=M=2 under SNR=20 dB and 0.38 compared with the Unaided Scheme, 0.33 compared with the Low-Complexity Scheme, and $1.5\times 10^{-5}$ compared with the SDR Scheme for N=4,M=2 under SNR=15 dB, which are shown in Table 2 and Table 3.

Accordingly, in comparison to the results in Figure 4 and the two tables, we can see that for the proposed FPP-SCA algorithm, the performance is always higher than the SDR-based one for different antennas and SNRs, and we can make the conclusion that our proposed FPP-SCA-based scheme performs better than the traditional SDR-based scheme.

More intriguingly, when N=M, the low-complexity scheme achieves a comparable performance to that of the FPP-SCA one and yields a better performance than that of N>M (e.g., 0.0 vs. 0.04 for SNR=30 dB shown in Table 2 and Table 3). Combined with the low complexity of the low-complexity scheme, it is more applicable than the FPP-SCA one in the N=M case. However, when N>M, in comparison to the FPP-SCA scheme, the performance of the low-complexity scheme is a bit worse owing to the influence of the enhancive diversity gain.

In summary, when the number of antennas at the relay node and source nodes are different, it is more beneficial to choose the FPP-SCA scheme.

**Figure 5:**
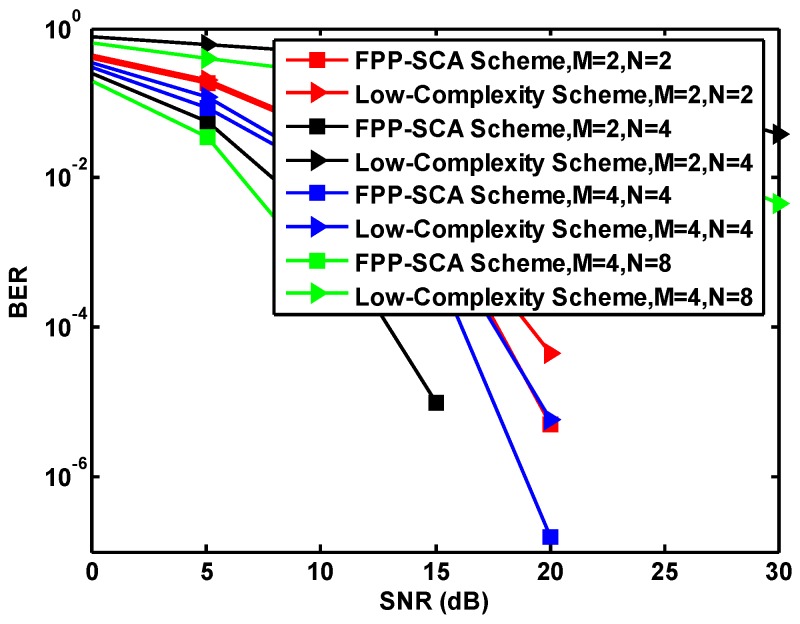
The antennas versus SNR for 50 iterations.

From Figure 5 and Table 4, we can see that the performance increases with the number of antennas for both schemes under M=N or N>M. When the number of antennas increases, more antennas can be used to suppress multipath fading with antenna diversity, to increase the system capacity, and to improve the performance. Considering the cost of computing of the low-complexity scheme and comparing the existing work proposed in Reference [35], we choose to use M=N=2 and M=2,N=4 for both schemes. In detail, in Table 4, $A^{M,N}$, $B^{M,N}$, $C^{M,N}$, and $D^{M,N}$ correspond to M=N=2; M=2,N=4; M=N=4; and M=4,N=8 for the FPP-SCA scheme and $E^{M,N}$, $F^{M,N}$, $G^{M,N}$, and $H^{M,N}$ correspond to M=N=2; M=2,N=4; M=N=4; and M=4,N=8 for the low-complexity scheme.

In order to verify the advantage of the proposed network, we make the comparison of our network and the existing BWSN proposed in Reference [35]. In Reference [35], a joint source and relay design for MIMO two-way relay networks with SWIPT considering a perfect CSI is proposed. In the network, the sources are equipped with PS receivers. The comparison is implemented under the same parameters for the two systems, and the results are as follows.

**Figure 6:**
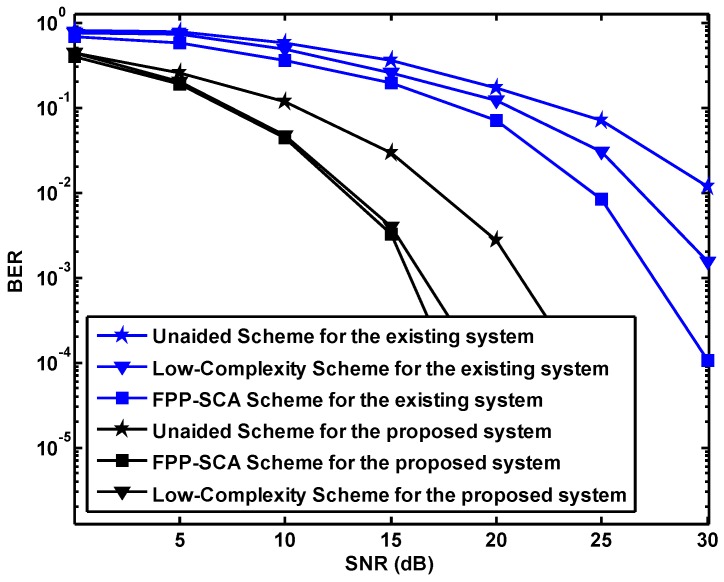
The comparison between the proposed network and existing BWSN N=M=2.

According to the results shown in Figure 6, it can be observed that the performance of the same algorithm based on the proposed system is preferred to that based on the BWSN, which verify the superiority of the proposed system.

## 5. Conclusions

In this paper, we have investigated the joint optimization problem for source and relay beamforming and source receiving in a MIMO FD BWSN SWIPT system. In terms of the problem, two iterative algorithms based on FPP-SCA and low-complexity diagonalizing designs which minimize the total-MSE subjected to the relay-and-source-transmitted power and energy-harvested constraints are proposed. The simulation results demonstrate that the low-complexity scheme always converges faster than the FPP-SCA based one, while the FPP-SCA-based scheme achieves a lower BER compared with the work of the low-complexity scheme. Moreover, when N=M, the performance of the low-complexity scheme yields better than that of N>M. In further works, we will analyze the system performance for multiple users and the interference suppression in the FD network scenario, where a large number of nodes are involved, and a discussion on the optimization scheme under the imperfect SCI will be developed.

## Figures and Tables

**Table 1:** The effects of β variation.

SNRs (dB)	FPP-SCA Scheme			Low-Complexity Scheme		
	β=0.3	β=0.5	β=0.7	β=0.3	β=0.5	β=0.7
**0**	0.35	0.25	0.2	0.80	0.77	0.74
**5**	0.12	0.06	0.03	0.66	0.62	0.59
**10**	0.01	0.003	9.3 × 10${}^{-4}$	0.52	0.47	0.45
**15**	1.8 × 10${}^{-4}$	1.0 × 10${}^{-5}$	0.0	0.38	0.33	0.31
**20**	0.0	0.0	0.0	0.25	0.22	0.19
**25**	0.0	0.0	0.0	0.14	0.11	0.09
**30**	0.0	0.0	0.0	0.06	0.04	0.03

**Table 2:** The BER performance for different schemes when M=N=2.

SNRs (dB)	Unaided Scheme	FPP-SCA Scheme	Low-Complexity Scheme	SDR Scheme
**0**	0.44	0.39	0.44	0.42
**5**	0.26	0.19	0.21	0.20
**10**	0.12	0.04	0.047	0.05
**15**	0.03	0.003	0.0043	0.004
**20**	0.0027	5.0 × 10${}^{-6}$	4.5 × 10${}^{-5}$	9.0 × 10${}^{-6}$
**25**	3.5 × 10${}^{-5}$	0.0	0.0	0.0
**30**	0.0	0.0	0.0	0.0

**Table 3:** The BER performance for different schemes when M=2,N=4.

SNRs (dB)	Unaided Scheme	FPP-SCA Scheme	Low-Complexity Scheme	SDR Scheme
**0**	0.80	0.25	0.77	0.28
**5**	0.67	0.06	0.62	0.07
**10**	0.52	0.003	0.47	0.005
**15**	0.38	2.5 × 10${}^{-5}$	0.33	4.0 × 10${}^{-5}$
**20**	0.27	0.0	0.22	0.0
**25**	0.18	0.0	0.11	0.0
**30**	0.12	0.0	0.04	0.0

**Table 4:** The effects of antennas variation.

SNRs (dB)	FPP-SCA Scheme				Low-Complexity Scheme			
	$A^{M,N}$	$B^{M,N}$	$C^{M,N}$	$D^{M,N}$	$E^{M,N}$	$F^{M,N}$	$G^{M,N}$	$H^{M,N}$
**0**	0.39	0.25	0.29	0.20	0.44	0.77	0.35	0.64
**5**	0.18	0.06	0.09	0.04	0.21	0.62	0.12	0.40
**10**	0.04	0.003	0.012	5.0 × 10${}^{-4}$	0.05	0.47	0.02	0.26
**15**	0.003	1.0 × 10${}^{-5}$	6.5 × 10${}^{-6}$	0.0	0.004	0.33	0.002	0.14
**20**	5.0 × 10${}^{-6}$	0.0	1.6 × 10${}^{-7}$	0.0	4.5 × 10${}^{-5}$	0.22	5.7 × 10${}^{-6}$	0.07
**25**	0.0	0.0	0.0	0.0	0.0	0.11	0.0	0.02
**30**	0.0	0.0	0.0	0.0	0.0	0.04	0.0	0.0045

## Abbreviations

The following abbreviations are used in this manuscript:

WSN	Wireless sensor network
MIMO	Multiple-input multiple-output
FD	Full-duplex
HD	Half-duplex
BWSN	Bidirectional wireless sensor network
BRN	Bidirectional relay network
OWRN	One-way relay network
OWSN	One-way wireless sensor network
SWIPT	Simultaneous wireless information and power transfer
TS	Time switching
PS	Power splitting
EH	Energy harvesting
ID	Information decoding
MSE	Mean square error
FPP-SCA	Feasible point pursuit-successive convex approximation
CP	Channel parallelization
BER	Bit error rate
GSVD	Generalized singular value decomposition
AF	Amplify and forward
DF	Decode and forward
AWGN	Additive white Gaussian noise
CSI	Channel state information
SNR	Signal noise ratio
SDR	Semidefinite relaxation
